# Prognostic impact of p53 and HER2 immunohistochemistry profiles in colorectal carcinoma

**DOI:** 10.3389/pore.2026.1612343

**Published:** 2026-05-14

**Authors:** Michal Grus, Hana Fišerová, Karolína Jelínková, Andrej Nikov, Radoslav Matěj, Petr Waldauf, Jan Hrudka

**Affiliations:** 1 Department of Pathology, 3rd Faculty of Medicine, Charles University, University Hospital Kralovske Vinohrady, Prague, Czechia; 2 Department of Surgery, 3rd Faculty of Medicine, Charles University, University Hospital Kralovske Vinohrady, Prague, Czechia; 3 Department of Pathology and Molecular Medicine, 3rd Faculty of Medicine, Charles University, Thomayer University Hospital, Prague, Czechia; 4 Department of Anaesthesia and Intensive Care Medicine, 3rd Faculty of Medicine, Charles University, University Hospital Kralovske Vinohrady, Prague, Czechia

**Keywords:** colorectal cancer, extraintestinal phenotype, HER2, immunohistochemistry, p53

## Abstract

Colorectal cancer (CRC) is a significant health issue worldwide, but represents biologically heterogeneous disease, necessitating improved characterization of molecular and immunophenotypic subgroups. In this retrospective study, we evaluated the prognostic significance and clinicopathological associations of p53 and HER2 expression in 290 surgically resected CRCs. Tissue microarrays from archival formalin-fixed paraffin-embedded samples were analyzed by immunohistochemistry. p53 expression was categorized into wild-type, overexpression, and null patterns, while HER2 was scored according to College of American Pathologists criteria used for gastric carcinoma. Survival analyses were performed using Kaplan–Meier estimates, univariable and multivariable Cox regression, and restricted mean survival time, and immunohistochemical results were correlated with established clinicopathological and molecular features. Neither aberrant p53 expression nor HER2 positivity had a significant impact on 10-year overall survival. The p53 null phenotype showed a significantly increased relative risk of death within 5 years and a significant adverse effect in multivariable Cox regression of 5-year survival adjusted for age. Aberrant p53 expression was significantly associated with left-sided tumor location, mismatch repair proficiency, CK20 positivity, and MUC6 negativity, consistent with the chromosomal instability pathway of CRC. HER2 expression was infrequent and showed no prognostic relevance; however, HER2-positive tumors were significantly associated with CK7 expression, supporting an “extraintestinal” immunophenotypic profile reminiscent of right-sided or non-intestinal differentiation. In conclusion, while p53 and HER2 expression lacked independent prognostic value, their distinct associations with tumor phenotype underscore biologically relevant CRC subgroups.

## Introduction

Colorectal cancer represents a significant global issue, as illustrated by the 1.9 million new cases and 880,000 deaths recorded in 2022 [1]. The majority of these tumors fall under the category of colorectal adenocarcinoma. Currently, there are multiple modalities of treatment available for the CRC, including surgery, chemotherapy, radiotherapy and targeted therapies utilizing antibodies. Nonetheless, the average 5-year survival rates for CRC in the US between 2014 and 2020 were 63% with notable differences between patients with localized CRC (91%) and those with distant metastatic CRC (13%) [2]. It is therefore crucial to identify new prognostic and predictive markers that could help us recognize the aggressive tumors better and treat them more efficiently. The p53 protein, often referred to as the “guardian of the genome”, coded by the *TP53* gene, plays a key role in regulating the cell cycle and acts as a major tumor suppressor. Its essential functions include DNA repair, inducing cell cycle arrest, cell senescence and promoting apoptosis [3]. In normal tissues, its cellular accumulation is prevented by negative regulators MDM2 and MDMX, which facilitate its ubiquitination and degradation [4]. Upon exposure to genotoxic stress, the p53 protein undergoes various post-translational modifications, including phosphorylation, acetylation, methylation, and glycosylation [5], which stabilize it and enable it to act as a transcription factor, influencing numerous signaling pathways [3]. While being a vital tumor suppressor gene, *TP53* is also the most frequently mutated one, with a mutational frequency of 35%–50% across all malignancies [5–8]. For some of these cancers (e.g., hepatocellular carcinoma, head and neck squamous cell carcinoma, acute myeloid leukemia or clear cell renal cell carcinoma), mutated *TP53* was reported as a prognostic marker [9]. In CRC, the reported mutational frequency ranges between 35% and 73% and its prognostic significance remains unclear [10]. Being frequently mutated and often having a negative prognostic impact, the p53 protein and its individual components have become targets for numerous therapeutic approaches. Currently, research is focused on drugs interacting with the MDM2/MDMX degradation pathway (RITA, ALRN-6924, RG7112, etc.), drugs restoring its wild-type function (APR-246, COTI-2, PRIMA-1, etc.), and drugs promoting mutant p53 degradation (ganetespib, onalespib, atorvastatin, etc.), among others [11, 12].

Human Epidermal growth factor Receptor 2 (HER2) is a transmembrane receptor with tyrosine kinase activity. Unlike other transmembrane receptors, it does not directly bind to a ligand but is activated instead through homodimerization or heterodimerization with other receptors from the EGFR family (EGFR, HER3, and HER4). HER2 homodimers and heterodimers activate specific signaling pathways (MAPK, PI3K/AKT, PKC, STAT1/STAT3) and promote cell growth and survival [13]. Consequently, its overexpression often induces the progression of various cancer types, e.g., breast, bladder, esophageal, gastric, cervical and gallbladder cancers [14–17]. Reportedly, the HER2 overexpression rate (IHC scores 2+ and 3+) is relatively low in CRC, without any negative impact on patient prognosis [18]. There are already studies investigating the effects of HER2-targeted therapy in a broad spectrum of solid tumors, including metastatic colorectal cancer, which have been showing meaningful clinical results [19–22]. However, additional data are necessary to draw more definitive conclusions. With each newly published study focusing on histological and molecular characteristics of CRC, it is becoming increasingly apparent that we are not talking about a singular tumor type but instead about groups of tumors, each with distinct prognoses and mutational patterns, with the most differences between left and right-sided CRC [23]. Yet, there still remain unanswered questions in understanding and effectively targeting the key driver mutations and achieving better treatment efficacy.

## Materials and methods

### Patient cohort

In this study, we retrospectively collected and analyzed data from 290 patients with histopathologically verified CRC, who underwent surgical treatment between 2010 and 2013 in the University Hospital Královské Vinohrady. Formalin-fixed paraffin-embedded (FFPE) samples of the tumor tissue from the patients included in this study were stored in the archive of the Department of Pathology. Follow-up data were obtained from the hospital medical records. The study was approved by the Ethical committee of University Hospital Královské Vinohrady, approval number VV/81/00/2026.

### Immunohistochemistry

We utilized a tissue microarray (TMA) method with the use of the 3DHistech TMA Master manual tissue arrayer. From each representative FFPE block containing tumor tissue, 2 cores were cut, each measuring 2 mm in diameter. The tumor area was manually annotated by a senior pathologist (JH) on the glass slide. Tissue microarray sampling was performed without regard to specific tumor regions—sampling was not aimed at the tumor center, invasive front, or similar area. Therefore, some patients had to be excluded during immunohistochemistry (IHC) evaluation due to lack of invasive CRC tissue in the TMA sample. Each recipient TMA block contained 20 tissue core samples from 10 patients. The IHC staining of the tissue sections was carried out using a Ventana BenchMark ULTRA autostainer (Ventana Medical Systems, Tucson, Arizona). The monoclonal antibodies against p53 (clone DO-7, dilution 1:250; BioSB, USA) and HER2 (clone 4B5; ready-to-use; Roche, Switzerland) were utilized. Visualization of the antibody reactions was done using the Ultraview Detection System (Ventana Medical Systems). HematoxylinII (Ventana, Roche Diagnostics; Cat. No. 05266726001) was used for counterstaining, and the slides were then dehydrated and covered in a xylene-based mounting medium.

### Immunohistochemistry (IHC) evaluation

**FIGURE 1:**
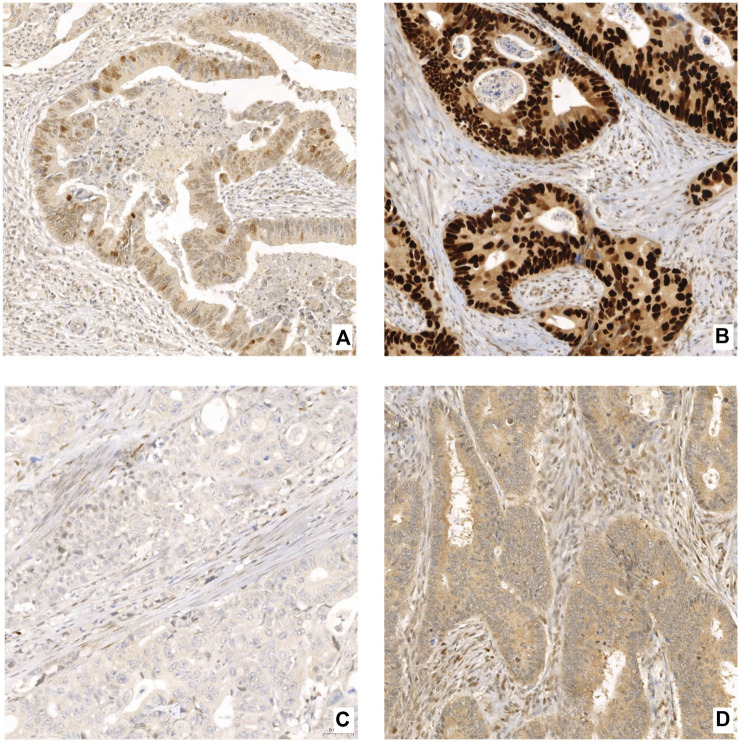
Examples of p53 IHC staining patterns (200 x): **(A)** wild type expression (nuclear staining of various intensities in tumor cells), **(B)** overexpression (strong nuclear staining in >80% of cancer cells), **(C)** null pattern (absence of p53 staining in both the nuclei and cytoplasm), **(D)** cytoplasmic expression (strong staining in the cytoplasm and absent in the nuclei).

**FIGURE 2:**
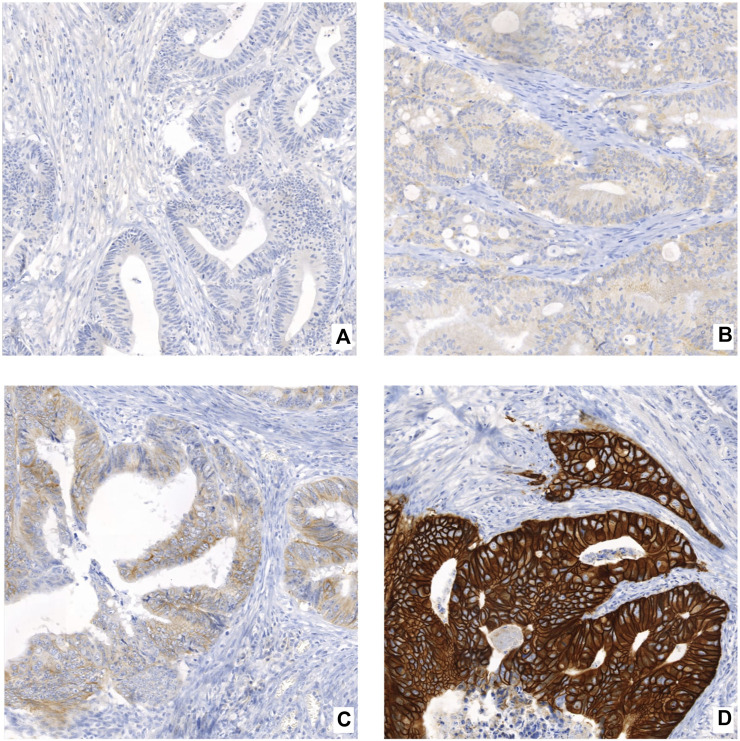
Examples of HER2 IHC scores in CRC tissue (200 x): **(A)** score 0 (no membrane staining or very weak or incomplete staining in ≤10% of tumor cells), **(B)** score 1+ (faint/barely perceptible membranous reactivity or incomplete membranous reactivity in >10% of tumor cells), **(C)** score 2+ (weak or moderate complete basolateral or lateral membrane reactivity in >10% of tumor cells), **(D)** score 3+ (strong complete, basolateral or lateral membrane reactivity in >10% of tumor cells).

Each slide was evaluated by two independent observers (MG and JH), experienced senior pathologist (JH) included, blinded to the follow-up and clinical data. For p53, four previously described distinct staining patterns were identified [24]. The wild-type pattern was defined by nuclear staining of various intensities in tumor cells (Figure 1A). The overexpression pattern was defined as strong nuclear staining in >80% of cancer cells (Figure 1B). The absence of p53 staining in both the nuclei and cytoplasm, accompanied by simultaneous wild-type staining in surrounding non-neoplastic tissue, was described as a null pattern (Figure 1C). If the strong staining was present in the cytoplasm and absent in the nuclei, the sample was classified as a cytoplasmic overexpression pattern (Figure 1D). For HER2, we applied a four-grade scoring system as commonly used in gastric cancer according to the guideline of the College of American Pathologists (CAP) [25]. Samples with no membrane staining or very weak or incomplete staining in ≤10% of tumor cells were classified as IHC score 0 (Figure 2A). IHC score 1+ was defined by faint/barely perceptible membranous reactivity or incomplete membranous reactivity in >10% of tumor cells (Figure 2B). IHC 2+ samples had weak or moderate complete basolateral or lateral membrane reactivity in >10% of tumor cells (Figure 2C). The samples with strong complete, basolateral or lateral membrane reactivity in >10% of tumor cells were classified as IHC 3+ (Figure 2D). In case of each of the two TMA samples from one patient classifying as different IHC scores, the higher score was preferred.

### Silver *in situ* hybridization

**FIGURE 3:**
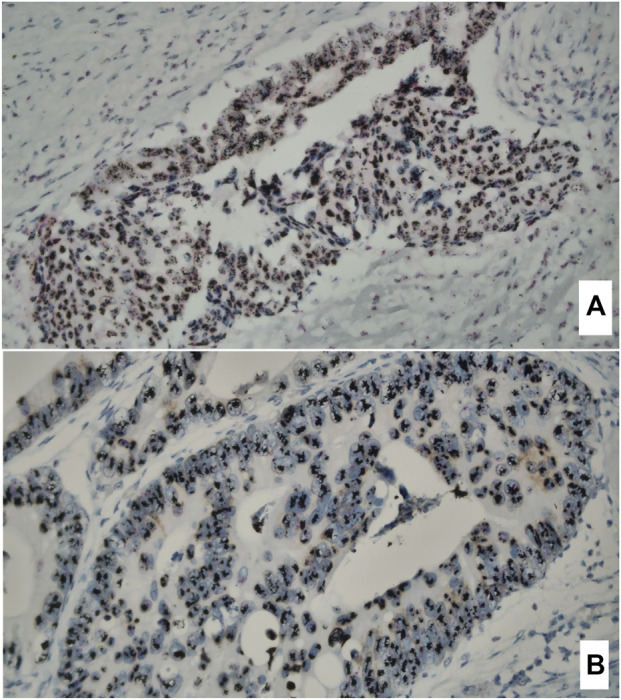
An assessment of HER2 gene copy number using the VENTANA Silver ISH (SISH) DNP Detection Kit. Twenty nuclei of invasive colorectal adenocarcinoma cells were evaluated with the following microscopic findings (400x): **(A)** A ratio of black signals (SISH) representing HER2 gene copies red signals (Red ISH Chr17) yielding a ratio of 7,15. The average number of black signals was 14.3 per cell. **(B)** A ratio of black signals (SISH) representing HER2 gene copies red signals (Red ISH Chr17) yielding a ratio of 11.6. The average number of black signals was 23.2 per cell. Both cases were evaluated as clear-cut HER2 amplification.

The cases with HER2 IHC score 3 and score 2 were further analyzed by silver *in situ* hybridization (SISH). Tissue sections (4–5 µm) were mounted on glass slides and processed using the VENTANA HER2 Dual ISH DNA Probe Cocktail on the BenchMark ULTRA automated staining platform (Roche Diagnostics, Mannheim, Germany), according to the manufacturer’s instructions. Following automated deparaffinization, heat-induced pretreatment and enzymatic digestion were performed to ensure optimal DNA accessibility. Tissue sections were co-hybridized with dual-color DNA probes targeting the HER2 gene locus and the centromere of chromosome 17 (CEP17). Post-hybridization stringency washes were followed by sequential chromogenic detection, yielding black silver *in situ* hybridization (SISH) signals for HER2 and red chromogenic *in situ* hybridization signals for CEP17. Slides were counterstained with hematoxylin and evaluated using standard bright-field light microscopy. The slides were evaluated by two experienced pathologists (JH and RM) following American Society of Clinical Oncology (ASCO)/CAP guidelines [25]. 20 non-overlapping, well-preserved invasive tumor nuclei were counted. The number of HER2 gene signals (black) and chromosome 17 centromere (CEP17) signals (red) was recorded for each nucleus. The HER2/CEP17 ratio was calculated (Figure 3).

### Statistics

The IHC results were correlated with 12 prognostic factors of CRC, including morphology type, grade, stage, site, mismatch repair (MMR) status, programmed death ligand 1 (PD-L1), CK7, CK20, SATB2, MUC4, MUC5AC, and MUC6, which have been previously published by our group, with methods described in detail elsewhere [26–28]. For overall survival (OS) analysis, Kaplan–Meier analysis was performed using the log-rank test and confidence intervals calculated by the log-log method, followed by restricted mean survival time (RMST) analysis. To calculate the hazard ratio (HR) for each parameter, univariable Cox regressions with 95% CIs were performed. A multivariable Cox regression analysis was performed, adjusting p53 and HER2 for age, binarized UICC stage (I + II vs. III + IV), and binarized histological grade (1 + 2 vs. 3). An analysis evaluating prognostic impact of p53 and HER2 profiles in UICC (I + II and III + IV) and grade (1 + 2 and 3) subgroups was performed. The survival analysis was truncated at 10 years and 5 years of follow-up time. As an additional analysis, relative risk (RR) was calculated for each group based on dichotomized 5-year survival (yes/no), presence of regional lymphonodal metastases (N1+2, and distant metastases (M1) at surgery. To analyse quantitative associations between variables, logistic regression/Pearson’s chi squared test was used. The variables were binarized as follows: localized tumors (UICC stage I + II) versus metastasizing tumors (UICC stage III + IV), low grade (grade 1 + 2) versus high grade (grade 3), right-sided tumors (cecum, ascending colon, hepatic flexure, transverse colon) versus left-sided tumors (splenic flexure, descending colon, sigmoid colon, rectum), MMR-deficient versus MMR-proficient, adenocarcinoma not otherwise specified (NOS) versus mucinous and signet ring carcinoma (clustered together). Additionally, immunohistochemistry markers (CK7, CK20, PD-L1, SATB2, MUC4, MUC5AC, MUC6) were binarized into positive and negative categories, as described in detail elsewhere [26–28]. We considered p-values ≤0.05 statistically significant. All statistical analyses were performed using R software, version 4.5.1.

## Results

The analyzed cohort consisted of 131 female and 159 male patients, with a mean age of 68 years at surgery. The clinical-pathological description of the cohort is summarized in Table 1. All research data are summarized in Supplementary Table S1. Eight patients were excluded from the p53 analysis and 22 patients were excluded from the HER2 analysis due to an insufficient amount of invasive carcinoma in both TMA cores. In one case, a p53 cytoplasmic overexpression pattern was identified. For statistical purposes, this case was classified as p53 overexpression. Three cases showed discordant p53 results between the two TMAs, with a wild-type pattern in one core and an overexpression pattern in the other; the overexpression pattern was used for the analysis. There were no instances of two different aberrant expression patterns appearing in one tumor. In five cases, one TMA core showed a HER2 score of 1 and the other a score of 0. In one case, one core showed a score of 2 and the other a score of 1. In these six cases, the higher HER2 score was used for analysis. All three cases with HER2 IHC score 3 were evaluated as HER2 amplified with HER2/CEP17 ratio ≥6.0 (Figure 3). From six cases with HER2 IHC score 2, one was evaluated as HER2 amplified with HER2/CEP17 ratio = 5.03 and average number of HER2 signals = 10.3. The remaining five cases did not meet the criteria for HER2 amplification.

**TABLE 1:** Clinical-pathological and immunohistochemistry description of the study cohort (n = 290).

Gender	
Male	159
Female	131
Status	
Alive	104
Dead of cancer	106
Dead of other cause/unknown cause	80
Site/laterality	
Right sided (coecum, ascendens, transversum)	112
Left sided (descendens, sigmoid, rectum)	178
UICC stage	​
I: T1–2 N0 M0	39
II: T3–4 N0 M0	106
III: any T, N+, M0	108
IV: any T, any N, M1	35
pT stage	
pT1	7
pT2	43
pT3	190
pT4	50
pN stage	
pN0	154
pN1	75
pN2	61
cM stage	
M0	255
M1	35
Histological grade	
G1	20
G2	190
G3	76
Unknown	4
Mismatch repair (MMR) status	
Proficient	257
Deficient	26
Unknown	7
Histological type	
Adenocarcinoma NOS	274
Mucinous carcinoma	14
Histological type	
Signet ring cell carcinoma	2
p53 immunohistochemistry status	
p53 wild type	120
p53 null phenotype	45
p53 overexpression	117
HER2 immunohistochemistry status	
HER2 score 0	213
HER2 score 1+	46
HER2 score 2+	6
HER2 score 3+	3
Cytokeratin 7 immunohistochemistry status	
CK7 positivity in ≥10% tumor cells	19
CK7 positivity in <10% tumor cells	263
Unknown	8
Cytokeratin 20 immunohistochemistry status	
CK20 positivity in ≥25% tumor cells	219
CK20 positivity in <25% tumor cells	64
Unknown	7
PD-L1 immunohistochemistry status	
PD-L1 positivity in ≥1% tumor cells	28
PD-L1 positivity in <1% tumor cells	250
Unknown	12
SATB2 immunohistochemistry status	
SATB2 positivity in ≥5% tumor cells	239
SATB2 positivity in <5% tumor cells	41
Unknown	10
MUC4 immunohistochemistry status	
MUC4 positivity in ≥10% tumor cells	87
MUC4 positivity in <10% tumor cells	188
Unknown	15
MUC5AC immunohistochemistry status	
MUC5AC positivity in >0% tumor cells	7
MUC5AC positivity in 0% tumor cells	281
Unknown	2
MUC6 immunohistochemistry status	
MUC6 positivity in >0% tumor cells	11
MUC6 positivity in 0% tumor cells	270
Unknown	9

**FIGURE 4:**
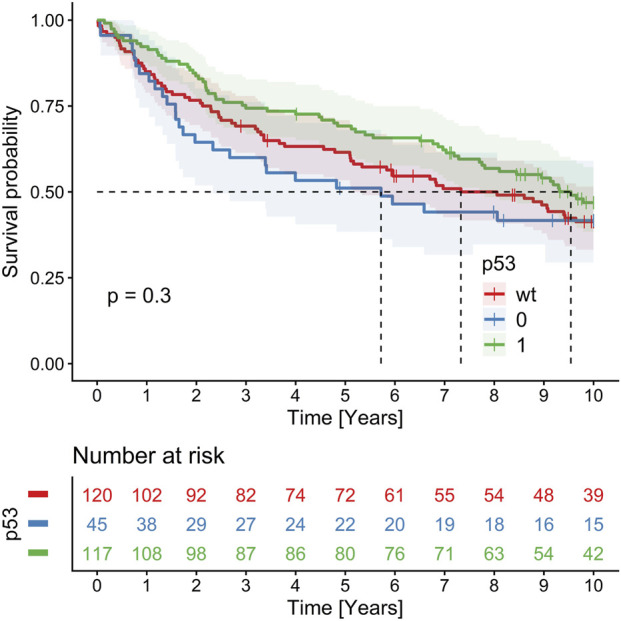
Kaplan-Meier 10-year OS analysis showing non-significant trend towards worse survival in CRCs with p53 null phenotype.

**FIGURE 5:**
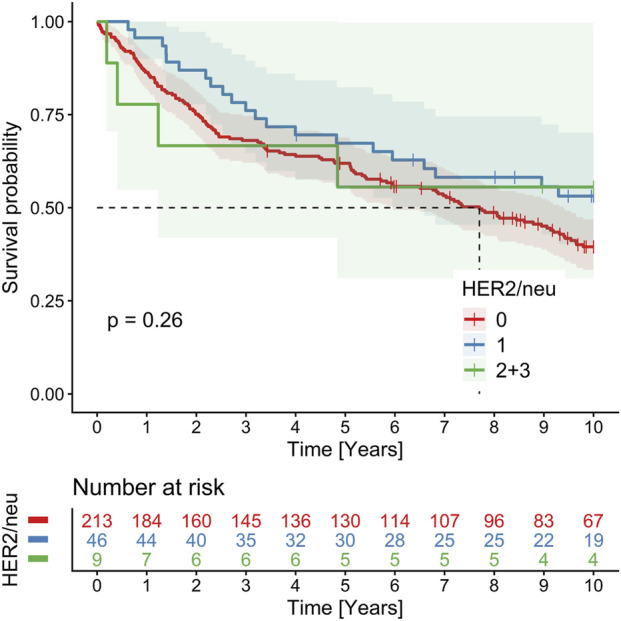
Kaplan-Meier 10-year OS analysis showing no prognostic impact of HER2 status in CRC.

**FIGURE 6:**
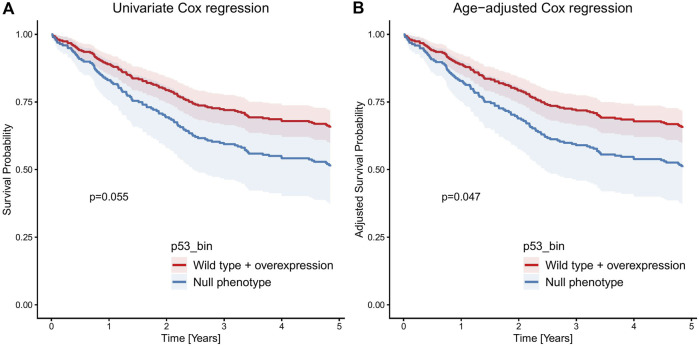
Cox regression 5-year OS analysis showing borderline significant negative effect of p53 null phenotype in the univariable **(A)**, and significant effect in the multivariable age-adjusted **(B)** analysis.

The numbers of cases for specific p53 and HER2 profiles are provided in Table 2. Results of Kaplan Meier 10 years OS analysis revealed no significant impact of p53 and HER2 profiles, these are summarized in Table 2. In Cox regression, there was no prognostic effect of p53 mutated profile [overexpression (n = 117) and null phenotype (n = 45) together] compared to p53 wild type (n = 120): HR = 0.87, 95% CIs 0.63–1.2, p = 0.4 (Figure 4), and p53 null phenotype separately (HR = 1.24, 95% CIs 0.82–1.90, p = 0.3). The same was true of HER2, if counted all positivity scores together (score 1 + n = 46, score 2 + n = 6, score 3 + n = 3) compared to HER2 negative cases (n = 213): HR = 0.70, 95% CIs 0.46, 1.08, p = 0.11 (Figure 5). Kaplan–Meier analysis and Cox regression censored at 5 years showed no significant impact of p53 or HER2 on OS, except for a borderline non-significant adverse effect of the p53 null phenotype (restricted mean OS = 3.40 vs. 3.91 years, p = 0.053; HR = 1.59, 95% CIs 0.99–2.54, p = 0.055) (Figure 6A), and significant negative effect of the p53 null phenotype in multivariable age-adjusted Cox regression (HR = 1.61, 95% CIs 1.01–2.58, p = 0.047) (Figure 6B).

**TABLE 2:** Kaplan Meier 10 years overall survival (OS) analysis results focusing on p53 and HER2 expression profiles in 290 cases of colorectal carcinoma.

Group	n =	Deaths	Restricted mean (rmean) OS (yrs)	Rmean standard error (yrs)	Median (yrs)	p value
p53 wild type	120	68	6.26	0.36	7.33	0.3
p53 null phenotype	45	26	5.64	0.60	5.72	
p53 overexpression	117	60	7.04	0.34	9.54	
HER2 score 0	213	125	6.26	0.27	7.70	0.26
HER2 score 1+	46	21	7.12	0.52	NA	
HER2 score 2–3+	9	4	6.30	1.44	NA	

Aberrant p53 expression had no significant impact on overall survival (OS) also in subgroup analyses: UICC stages I–II (n = 147; HR 0.80, 95% CI 0.48–1.32; p = 0.4), stages III–IV (n = 143; HR 0.87, 95% CI 0.58–1.31; p = 0.5), grade 1 + 2 tumors (n = 209; HR 0.85, 95% CI 0.58–1.25; p = 0.4), and grade 3 tumors (n = 76; HR 0.88, 95% CI 0.48–1.62; p = 0.7). Similarly, HER2 positivity (scores 1 + 2+3) showed no significant association with OS across subgroups: UICC stages I–II (n = 147; HR 0.59, 95% CI 0.30–1.17; p = 0.13), stages III–IV (n = 143; HR 0.87, 95% CI 0.50–1.51; p = 0.6), grade 1 + 2 tumors (n = 209; HR 0.64, 95% CI 0.39–1.07; p = 0.089), and grade 3 tumors (n = 76; HR 0.91, 95% CI 0.40–2.05; p = 0.8). Neither p53 nor HER2 achieved independent prognostic significance after adjustment for age, UICC stage or tumor grade in the multivariable Cox regression. Only HER2 score 0 cases displayed slightly (non-significantly) shorter OS if adjusted for patient´s age or tumor grade (Table 3).

**TABLE 3:** Multivariable Cox regression of 10 years OS adjusting p53 and HER2 for age, binarized UICC stage and binarized histological tumor grade.

Group	Characteristic	HR	95% CIs	p-value
p53 adj. for age	p53 wild type	1.12	0.82–1.54	0.5
	Age	1.03	1.01–1.04	0.001
p53 adj. for UICC	p53 wild type	1.19	0.86–1.64	0.3
	UICC III + IV	2.09	1.51–2.89	<0.001
p53 adj. for grade	p53 wild type	1.18	0.84–1.64	0.4
	Grade 3	1.11	0.76–1.64	0.6
HER2 adj. for age	HER2 score 0	1.47	0.95–2.22	0.084
	Age	1.02	1.01, 1.04	0.008
HER2 adj. For UICC	HER2 score 0	1.35	0.88–2.08	0.2
	UICC III + IV	2.05	1.48–2.85	<0.001
HER2 adj. for grade	HER2 score 0	1.45	0.93–2.27	0.093
​	Grade 3	1.09	0.73–1.61	0.7

The RR analysis of 5-year survival (yes/no) revealed no significant impact of p53 aberrant profile (RR = 0.94, 95% CIs 0.69–1.28, p = 0.69), HER2 score 1 + 2+3 (RR = 0.92, 95% CIs 0.61–1.37, p = 0.67), and HER2 score 2 + 3 (RR = 1.19, 95% CIs 0.56–2.50, p = 0.66); but borderline non-significant effect of p53 overexpression (RR = 1.35, 95% CI 0.98–1.89, p = 0.07). There was a significant RR in p53 null phenotype (RR = 1.43, 95% CIs 1.01–2.01, p = 0.042). In RR analysis focusing on staging, HER2 positivity (1 + 2+3) showed a non-significant trend towards a lower risk of nodal metastases (RR = 0.35, 95% CIs 0.11–1.07, p = 0.064) and distant metastases (RR = 0.40, 95% CIs 0.13–1.27, p = 0.119). In contrast, p53 status (aberrant vs. wild-type) was not significantly associated with nodal (RR = 1.27, 95% CIs 0.73–2.19, p = 0.399) or distant metastases (RR = 0.98, 95% CIs 0.52–1.84, p = 0.954). Evaluating HER2 score 1+, HER2 scores 2+/3+, p53 overexpression, and the p53 null phenotype, no significant associations were observed between RR and either lymph node or distant metastases. There were only non-significant trends towards nodal metastases for p53 null phenotype (RR = 1.42, 95% CIs 0.90–2.24, p = 0.13); and in HER2 score 2 + 3, trend towards lower risk of nodal (RR = 0.41, 95% CIs 0.13–1.28, p = 0.12), and distant (RR = 0.39, 95% CIs 0.11–1.37, p = 0.14) metastases.

Logistic regression/Pearson’s chi-squared test revealed several significant associations between p53 and HER2 immunohistochemistry profiles with previously described variables. CRCs with p53 mutated (aberrant) profiles were prone to be left sided (Odds ratio/OR = 2.44, 95%CIs = 1.45–4.12, p < 0.001), MMR proficient (OR = 12.2, 95%CIs = 3.53–65, p < 0.001), CK20 positive (OR = 2.26, 95%CIs = 1.23–4.19, p = 0.006), and MUC6 negative (OR = 6.25, 95%CIs = 1.30–50, p = 0.01). Tumors with HER2 positivity (scores 1–3+ together) were more often CK7 positive (OR = 3.29, 95%CIs = 0.99–10.5, p = 0.026). No associations of p53 or HER2 were found with PD-L1, SATB2, MUC4, or MUC5AC.

## Discussion

Our study focused on the prognostic implications and quantitative associations of p53 and HER2 profiles examined by immunohistochemistry in CRC. Mutated p53 profile revealed no prognostic impact, but was tightly associated with left sided tumors, MMR proficient status, CK20 positivity and MUC6 negativity. CRC was used as a model tumor in the theory of gradual acquisition of oncogenic mutations in the canonical Vogelstein cancerogenesis model [29]. *TP53* is mutated in approximately one half of CRCs according to the Cancer Genome Atlas Network [30]. 20 years ago, Munro et al. conducted a meta-analysis comprising 168 studies on 18766 patients with CRC, using a very heterogeneous methodology. In this meta-analysis, aberrant p53 status was associated with a modest but statistically significant increase in the risk of death (relative risk [RR] 1.32 for immunohistochemistry and 1.31 for mutation analysis). It should be noted that RR reflects cumulative event probabilities at a fixed time point and does not account for time-to-event dynamics, in contrast to hazard ratios (HR) derived from survival analysis. To facilitate comparison with these findings, we calculated RR for 5-year survival in our cohort based on immunohistochemical p53 status. No significant association was observed (RR = 0.94, 95% CI 0.69–1.28, p = 0.69), which is consistent with the overall modest and variable prognostic impact of p53 reported in the literature. Of note, we observed significantly higher RR in p53 null phenotype (RR = 1.43, 95% CIs 1.01–2.01, p = 0.042) and a negative prognostic effect of p53 null phenotype in the multivariable age-adjusted Cox regression (HR = 1.61, 95% CIs 1.01–2.58, p = 0.047). In Munro´s analysis, p53 emerged as a questionable predictor of postoperative metastasis occurrence; RR = 0.92 (95% CI 0.6–1.39) by immunohistochemistry and RR = 1.67 (95% CI 1.21–2.30) by molecular analysis. Likewise, according to this meta-analysis, the role of p53 as a predictor of chemotherapy response remains questionable [31]. To date, there have been numerous studies focusing on the prognostic role of p53 in CRC. Our findings are consistent with the aforementioned meta-analysis, probably due to the abundance of p53-mutated CRCs and the high complexity of molecular CRC pathogenesis. However, many older studies are limited by methodological issues, discerning p53 profiles as positive or negative, without considering mutated and wild-type immunohistochemistry patterns. Interestingly, Maki et al. recently described, in a large cohort (n = 458) of patients with surgically resected CRC liver metastases, an association between *TP53*-mutated tumors and poorer response to chemotherapy as well as shorter OS [32]. An interesting observation is shorter OS and poorer chemotherapy response in CRC patients with complete loss of p53, i.e., a null immunohistochemical phenotype, compared to p53 wild type and p53 overexpression [33]. This can be explained by chemotherapy-induced activation of apoptosis in tumor cells with functional p53, while in patients with p53 overexpression, apoptosis is at least partially mediated by the non-mutated *TP53* allele. This is in line with our finding of a significantly smaller proportion of 5-year survivors in p53 null phenotype CRCs, and significantly worse survival of those in the 5-year age-adjusted Cox regression. A similar trend as in our results is suggested by a meta-analysis of metastatic CRC, where detected *TP53* mutations were strongly associated with left-sided localization and with a non-significant trend towards worse OS (HR = 1.3, 95% CI 0.75–2.25, p = 0.3) [10]. To date, three major molecular pathways of colorectal carcinogenesis have been described: chromosomal instability, microsatellite instability (MSI), and the serrated pathway, the latter being closely associated with the CpG island methylator phenotype (CIMP) [34]. The chromosomal instable pathway is the most common, it is prone to be sporadic (non-hereditary) localized in the distal colon or rectum. *TP53* is one of the canonical driver mutations in this sporadic type of CRC, which is in line with the newly described association of “left side predominant” expression of CK20 [26, 35] and negative association with “right side predominant” or “extraintestinal” MUC6-expression [36].

As is broadly known, HER2 is a negative prognostic marker in breast cancer [37]. The newly identified association of HER2 and CK7 expression suggests that HER2 may be part of “extraintestinal features”, which include right-sided localization, CK7 positivity, and a similar prognosis to i.e., pulmonary adenocarcinoma, which constantly express CK7 [26]. However, we did not prove the prognostic impact of HER2 expression in a relatively large cohort, probably due to the overall paucity of HER2 overexpression in CRC. The subgroup analysis and multivariable Cox regression showed a borderline non-significant trend towards better survival in patients with low grade HER2-positive tumors (p = 0.089). Given the small number of HER2-positive cases overall (n = 55 with score 1 + 2+3), this finding should be interpreted cautiously and does not support a definitive conclusion, but may warrant verification in larger cohorts stratified by grade. HER2 overexpression at the immunohistochemistry level is present in 1.4%–15% of CRC [38–42], depending on scoring methodology, with slight rectal and left sided predominance [40]. Studies with similar cohorts focusing on prognostic implications of HER2 rendered similar results to ours with sparse HER2 overexpressed cases and no significant prognostic impact [43]. Quingguo et al. reported an association of HER2 expression with tumor size and distant metastasis, without impact on OS [41]. Park et al. described a tendency for higher rates of nodal metastasis, poor mean survival and 5-year survival in HER2 overexpressed CRCs in a relatively small dataset [44]. Heppner et al. described a negative prognostic effect in a large study only on HER2 score 3+ [45]. On the other hand, Condradi et al. described the beneficial prognostic impact of HER2 in patients with CRC who underwent neoadjuvant treatment [46]. The inconsistency among studies may be attributable to preanalytical and laboratory factors as fixation methods, antigen retrieval and incubation time, various antibody clones and different scoring methodologies. Also, clinical factors such as neoadjuvant treatment may influence HER2 expression and survival analysis results [45]. Larger study cohorts using rigorous methodology with higher numbers of HER2 3+ cases are warranted. Nevertheless, clinical studies showed robust correlation between HER2 amplification and clinical response to ERBB2-targeted therapy in metastatic colorectal cancer [47–49]. Although HER2 appears to have limited prognostic significance in colorectal cancer, it may have a primarily predictive role, as demonstrated in the HERACLES trial, in which patients with HER2-positive metastatic CRC benefited from dual anti-HER2 therapy with trastuzumab and lapatinib [22, 47, 50].

A potential limitation of this study is the use of the TMA technique and the associated risk of sampling error in case of tumor heterogeneity. However, several studies have demonstrated good concordance between immunohistochemical profiles across different tissue microarray cores in colorectal carcinoma, including our own work [28] and a study specifically analyzing p53 in TMA samples in CRC [51].

## Conclusion

In our CRC cohort, the p53 “mutated” immunohistochemical pattern had no independent prognostic value, but was associated with left-sided location, MMR proficiency, CK20 positivity, and MUC6 negativity - features consistent with the chromosomal-instability pathway. Findings of worse outcomes in the p53-“null” IHC phenotype highlight the importance of separating null, overexpression, and wild-type patterns in future analyses. We also observed an association between HER2 and CK7 expression, supporting an “extraintestinal/right-sided” immunophenotypic subset; however, HER2 showed no prognostic impact in our series, likely reflecting its low prevalence, although HER2 expression is a very important target for innovative therapeutic modalities. The novelty of our study lies mainly in: (1) the long-term assessment of 10-year overall survival in a uniform surgical cohort, (2) the interpretation of p53 at the level of multiple IHC patterns rather than a simple binary positive/negative classification, documenting negative prognostic impact of p53 null phenotype in 5 years analysis, and (3) the integration of p53/HER2 with a broader immunophenotype (CK7/CK20/MUC6, etc.) and with the concept of biologically distinct CRC subgroups.

## Data Availability

The original contributions presented in the study are included in the article/Supplementary Material, further inquiries can be directed to the corresponding author.
